# Effects of Resveratrol on the Renin-Angiotensin System in the Aging Kidney

**DOI:** 10.3390/nu10111741

**Published:** 2018-11-12

**Authors:** In-Ae Jang, Eun Nim Kim, Ji Hee Lim, Min Young Kim, Tae Hyun Ban, Hye Eun Yoon, Cheol Whee Park, Yoon Sik Chang, Bum Soon Choi

**Affiliations:** 1 Department of Internal medicine, College of Medicine, The Catholic University of Korea, Seoul 06591, Korea; inae623@hanmail.net (I.-A.J.); deux0123@catholic.ac.kr (T.H.B.); berrynana@catholic.ac.kr (H.E.Y.); cheolwhee@catholic.ac.kr (C.W.P.); ysc543@unitel.co.kr (Y.S.C.); 2 Division of Medical Cell Biology, Department of Biomedical Science, College of Medicine, The Catholic University of Korea, Seoul 06591, Korea; kun0512@hanmail.net (E.N.K.); didsuai@hanmail.net (J.H.L.); sweetshow@naver.com (M.Y.K.); 3 Division of Nephrology, Department of Internal Medicine, Seoul St. Mary’s Hospital, Seoul 06591, Korea; 4 Division of Nephrology, Department of Internal Medicine, Incheon St. Mary’s Hospital, Incheon 21431, Korea; 5 Division of Nephrology, Department of Internal Medicine, Yeouido St. Mary’s Hospital, Seoul 07345, Korea; 6 Division of Nephrology, Department of Internal Medicine, St. Paul’s Hospital, Seoul 02559, Korea

**Keywords:** renin-angiotensin system, angiotensin converting enzyme 2, kidney, resveratrol, aging

## Abstract

The renin-angiotensin system (RAS), especially the angiotensin II (Ang II)/angiotensin II type 1 receptor (AT1R) axis, plays an important role in the aging process of the kidney, through increased tissue reactive oxygen species production and progressively increased oxidative stress. In contrast, the angiotensin 1-7 (Ang 1-7)/Mas receptor (MasR) axis, which counteracts the effects of Ang II, is protective for end-organ damage. To evaluate the ability of resveratrol (RSV) to modulate the RAS in aging kidneys, eighteen-month-old male C57BL/6 mice were divided into two groups that received either normal mouse chow or chow containing resveratrol, for six months. Renal expressions of RAS components, as well as pro- and antioxidant enzymes, were measured and mouse kidneys were isolated for histopathology. Resveratrol-treated mice demonstrated better renal function and reduced albuminuria, with improved renal histologic findings. Resveratrol suppressed the Ang II/AT1R axis and enhanced the AT2R/Ang 1-7/MasR axis. Additionally, the expression of nicotinamide adenine dinucleotide phosphate oxidase 4, 8-hydroxy-2′-deoxyguanosine, 3-nitrotyrosine, collagen IV, and fibronectin was decreased, while the expression of endothelial nitric oxide synthase and superoxide dismutase 2 was increased by resveratrol treatment. These findings demonstrate that resveratrol exerts protective effects on aging kidneys by reducing oxidative stress, inflammation, and fibrosis, through Ang II suppression and MasR activation.

## 1. Introduction

Aging causes progressive deterioration of organs, leading to impaired tissue function, increased vulnerability to stress, and death [1,2]. The kidney is one of the most susceptible target organs of age-associated tissue damage [3], and the high incidence of chronic kidney disease, in the elderly, is a health problem, worldwide [4,5].

Various processes are involved in the deterioration seen in aging kidneys, including oxidative stress, mitochondrial dysfunction, inflammation, altered calcium regulation, and activation of the renin-angiotensin system (RAS) [6,7]. The RAS modulates cell growth and senescence by activating the classic RAS axis—angiotensin converting enzyme (ACE)/Ang II/AT1R. This axis suppresses pro-survival genes and enhances reactive oxygen species (ROS) and pro-inflammatory cytokines production, resulting in chronic inflammation and cell senescence [8,9]. In contrast, the ACE2/Ang 1-7/MasR axis acts as a counter-regulator of classic Ang II-mediated effects [10,11,12].

Resveratrol is a polyphenolic phytoalexin that exists naturally in various plant parts and products, such as grapes, red wine, berries, and peanut skins, and has numerous beneficial health effects [13,14]. It acts as an activator of silent information regulator 1 (SIRT1) [15,16], exerting anti-senescent effects, as well as antioxidant and anti-inflammatory activities [17,18]. We previously reported that resveratrol exerts renal protective effect by activating Nrf2 and SIRT1 signaling pathways [19]. In this study, we hypothesized that resveratrol would attenuate the aging process in the kidney of mice by modulating RAS components, especially by the activation of the ACE2/Ang 1-7/MasR axis, and furthermore, we examined pro-inflammatory and antioxidant molecular changes in kidneys of aging mice.

## 2. Materials and Methods

### 2.1. Study Design and Animals

Eighteen-month-aged male C57BL/6 mice were purchased from the Korea Research Institute of Bioscience and Biotechnology (Chungcheongbuk-do, Korea). The Animal Care Committee of the Catholic University of Korea approved the experimental protocol and the experiments were performed in accordance with our institutional animal care guidelines. Mice were housed in a controlled temperature and controlled light environment with 12:12-h light-dark cycles and had free access to water. The aged male C57BL/6 mice were divided into two groups: The control group (*n* = 7) received normal mice chow (PicoLab Rodent Diet 20 5053, Labdiet, St. Louis, MO, USA) and the resveratrol-treated group (*n* = 7) received normal mice chow mixed with resveratrol (40 mg/kg, Sigma, St. Louis, MO, USA). The food was changed every 24 h and a calculated regular amount of food was fed daily, for a six month period. The experimental conditions were set with reference to the article of Baur et al. [20]. The mice were sacrificed at the age of twenty-four month.

### 2.2. Evaluation of the Renal Function

Mice were placed in individual mouse metabolic cages (Tecniplast, Gazzada, Italy) with access to water and food for 24 h. Urine collection was done every four weeks and data from month 18 and 24 was used in this experiment. Albuminuria (Albuwell M, Exocell, Philadelphia, PA, USA) and urine creatinine concentration (The Creatinine Companion, Exocell, Philadelphia, PA, USA) were measured using ELISA kits. Serum creatinine concentrations and Blood urea nitrogen were measured using i-STAT system Cartridges (CHEM8+, Abbott Point of Care, Abbott Park, IL, USA). Creatinine clearance was calculated using a standard formula (urine creatinine (mg/dL) × urine volume (mL/24 h)/serum creatinine (mg/dL) × 1440 (min/24 h)).

### 2.3. Histological Assessment of the Renal Tissue

Kidney tissue samples were fixed in 10% formalin. The tissues were embedded in low-temperature melting paraffin, and 4 microns, thick sections were processed and stained with a periodic acid–Schiff (PAS), and Masson’s trichrome. The glomerular volume and mesangial matrix were quantified for kidney tissue cross-sections, using PAS staining. The relative mesangial area was expressed as mesangial/glomerular surface area. A finding of tubulointerstitial fibrosis was defined as a matrix-rich expansion of the interstitium in Masson’s trichrome. Ten randomly selected fields, per section, were assessed. All of these sections were examined in a blinded manner, using a color-image analyzer (TDI Scope Eye, Version 3.5 for Windows, Olympus, Tokyo, Japan) and quantified using image J (Wayne Rasband national institutes of health, Bethesda, MD, USA).

### 2.4. Immunohistochemistry

Immunohistochemistry was performed to determine changes of Ang II, angiotensin target receptors (AT1R, AT2R and MasR) and oxidative stress using 8-hydroxy-deoxyguanosine (8-OHdG) and 3-nitrotyrosine. Paraffin sections were deparaffinized in xylene and hydrated in ethanol, before staining, and treated with an antigen-unmasking solution, consisting of 10 mM sodium citrate, pH 6.0 and then washed with phosphate-buffered saline. Sections were incubated with 3% H_2_O_2_, in methanol, to block the endogenous peroxidase activity. Nonspecific binding was blocked with 10% normal horse serum. After incubating with the primary antibody to Ang II, AT2R and MasR (Novus Biologicals, Littleton, CO, USA), AT1R and 3-nitrotyrosine (Santa Cruz Biotechnology, Dallas, TX, USA), and 8-OHdG (Japan Institute for the Control of Aging, Shizuoka, Japan), at 4 °C, overnight, antibodies were visualized with a peroxidase conjugated secondary antibody, using the Vector Impress kit (Vector Laboratories, Burlingame, CA, USA). Sections were then dehydrated in ethanol, cleared in xylene and mounted without counterstaining. All sections were assessed using a color-image analyzer (TDI Scope Eye, Version 3.5 for Windows, Olympus, Tokyo, Japan).

### 2.5. Western Blot Analysis

Total protein was extracted from the kidney tissues, using Pro-Prep Protein Extraction Solution (Intron Biotechnology, Gyeonggi-Do, Korea), according to the manufacturer’s instructions. Extracted protein was subjected to SDS-polyacrylamide gel electrophoresis and transferred onto a Nitrocellulose membrane (Amersham Biosciences, Amersham, UK). Membranes blocked with 3% nonfat milk in Tris-buffered saline (TBS), containing 0.1% Tween-20 for 1 h, at room temperature. Then membranes were incubated overnight, at 4 °C, with primary antibodies in 3% nonfat milk or 3% BSA in Tris-buffered saline (TBS), containing 0.1%. After that, the membranes were washed in TBS containing 0.1% Tween-20 and were then incubated with peroxidase-conjugated secondary antibody, for 2 h, at room temperature. Immunoreactive bands were detected using Amersham ECL Prime Western Blotting Detection Reagent (Amersham Biosciences, Amersham, UK). Western blot analysis was performed using the following antibodies—transforming growth factor-β (TGF-β, R&D Systems, Minneapolis, MN, USA), collagen IV (Abcam, Cambridge, UK), fibronectin (Proteintech Group Inc, Chicago, IL, USA), ACE (Santa Cruz Biotechnology, Dallas, TX, USA), ACE2 (R&D Systems, Minneapolis, MN, USA), AT1R (Santa Cruz Biotechnology, Dallas, TX, USA), AT2R (Novus Biologicals, Littleton, CO, USA), prorenin receptor (PRR, Sigma life science, St. Louis, MO, USA), MasR (Novus Biologicals, Littleton, CO, USA), endothelial nitric oxide synthase (eNOS, Cell Signaling Technology Inc., Beverly, MA, USA), phosphorylated (phospho)-Ser1177 eNOS (Cell Signaling Technology Inc., Beverly, MA, USA), Nicotinamide adenine dinucleotide phosphate oxidase 2 (NOX2, BD Biosciences, Mountain View, MD, USA), Nicotinamide adenine dinucleotide phosphate oxidase 4 (NOX4, Santa Cruz Biotechnology, Dallas, TX, USA), superoxide dismutase 1 (SOD1, Enzo Life Sciences, Farmingdale, NY, USA), superoxide dismutase 2 (SOD2, Abcam, Cambridge, UK), and β-actin (Sigma Life Science, St. Louis, MO, USA).

### 2.6. Enzyme Immunoassay

Levels of Ang II and Ang 1-7 in Serum and renal tissue homogenates were measured using competitive enzyme immunoassay (Cusabio Biotech Co., Wuhan, China), according to the manufacturer’s protocols.

### 2.7. Statistical Analysis

Data are expressed as means ± standard deviation (SD). Differences between the groups were examined for statistical significance, using ANOVA and unpaired *t*-test (SPSS v. 19.0, IBM, Armonk, NY, USA). *p* values of less than 0.05 were considered significant.

## 3. Results

### 3.1. Effects of Resveratrol on Renal Function in Aging Mice

**Figure 1:**
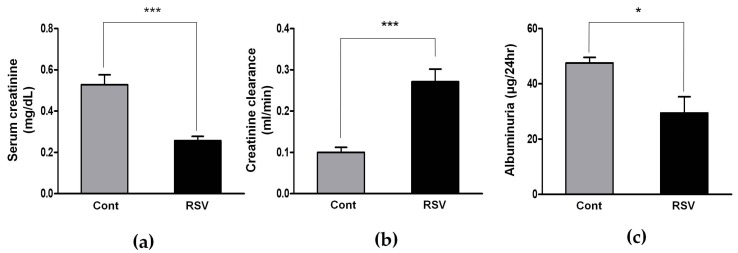
Effects of resveratrol on renal function of eighteen-month-old male C57BL/6 mice. Compared to the control group, resveratrol group showed (**a**) lower serum creatinine, (**b**) increased creatinine clearance, and (**c**) reduced 24 h albuminuria (* *p* < 0.05, *** *p* < 0.001).

Changes in renal function of aging mice were measured, before and after resveratrol treatment, and the results were compared with control mice. Serum creatinine was decreased in resveratrol-treated mice, compared with the control group mice (control 0.53 ± 0.05 vs. resveratrol 0.25 ± 0.02 mg/dL, Figure 1a). Creatinine clearance was significantly greater in the resveratrol-treated group than the control group (control 0.10 ± 0.01 vs. resveratrol 0.27 ± 0.03 mL/min, Figure 1b). Twenty-four hour albuminuria was significantly decreased in the resveratrol-treated group, compared with the control group (control 47.60 ± 1.97 vs. resveratrol 29.47 ± 5.85 μg/24 h, Figure 1c). These results show that resveratrol reduces albuminuria and, thus, improves kidney function in aging mice.

### 3.2. Effects of Resveratrol on the Renal Histological Changes in Aging Mice

**Figure 2:**
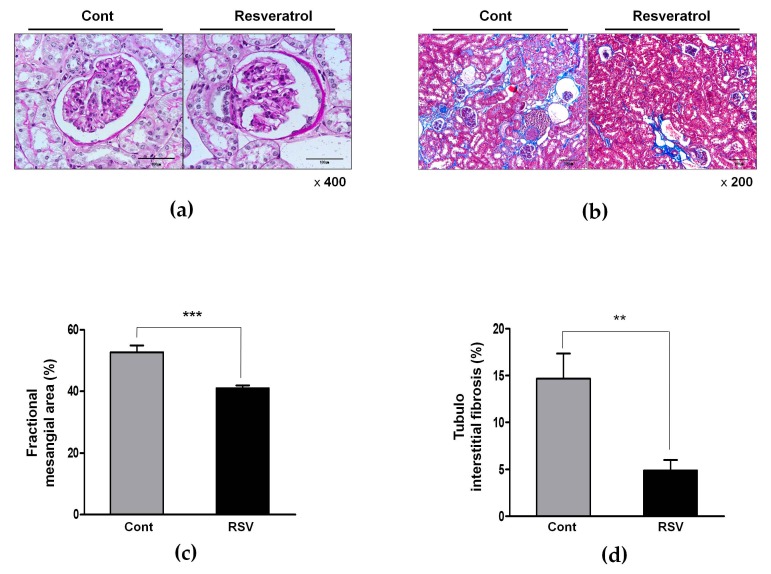
Effects of resveratrol on aging-related histological renal injury. (**a**) Representative photomicrographs of the periodic acid–Schiff-(PAS)-stained kidney showed less expansion of the mesangial area in the RSV group (original magnification 400×). (**b**) Representative sections of the Masson’s trichrome-stained kidney showed significantly less tubulointerstitial fibrosis in the RSV group (original magnification 200×). (**c**) Quantitative assessments of the areas of extracellular matrix in the glomerulus. (**d**) Quantitative assessment of the areas of tubulointerstitial fibrosis in the control and RSV groups (** *p* < 0.01, *** *p* < 0.001).

Histological examination demonstrated that the fractional mesangial area was reduced in the resveratrol-treated group, as compared to the control group (control 52.75 ± 2.18 vs. resveratrol 41.03 ± 0.92%, Figure 2a,c). Additionally, tubulointerstitial fibrosis was substantially less in the resveratrol-treated group than in the control group (control 14.68 ± 2.69 vs. resveratrol 4.89 ± 1.11%, Figure 2b,d). Thus, renal histological deterioration induced by aging was improved by resveratrol treatment.

### 3.3. Resveratrol Inhibits the Ang II/AT1R Axis in Aging Mice

**Figure 3:**
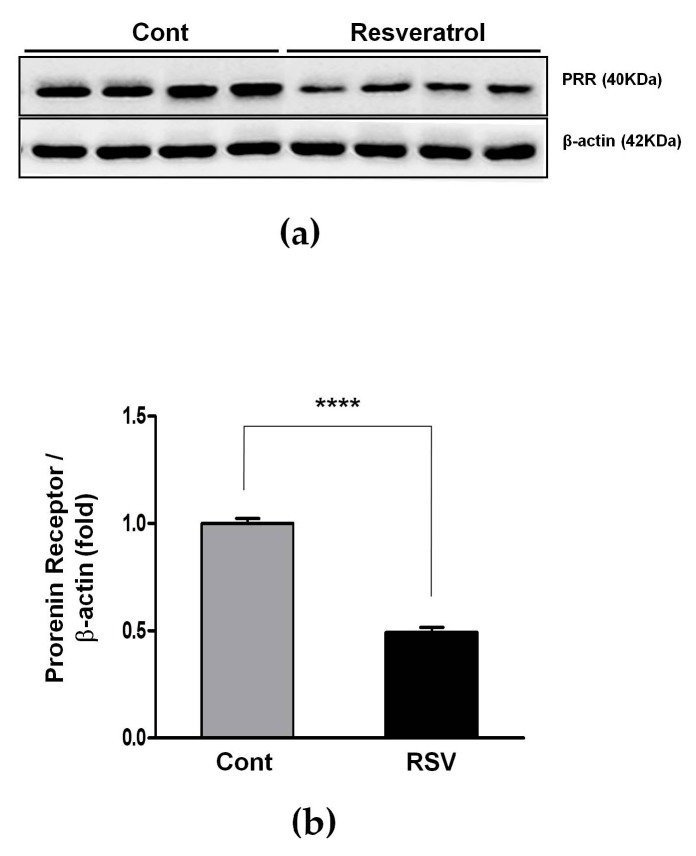
Effects of resveratrol on the expression of prorenin receptors. (**a**) Representative western blot analysis of prorenin receptors expression. (**b**) Prorenin receptors levels were decreased in the RSV group. Quantitative analysis of the results is shown (**** *p* < 0.0001).

The RAS plays an important role in the aging process of kidneys, through increased tissue ROS production and progressively increased oxidative stress. Expression of the PRR, ACE, and Ang II was measured by western blot analyses. Expression of the PRR decreased significantly in the resveratrol-treated group, compared with the control group (control 1.00 ± 0.02 vs. resveratrol 0.49 ± 0.02-fold, Figure 3).

**Figure 4:**
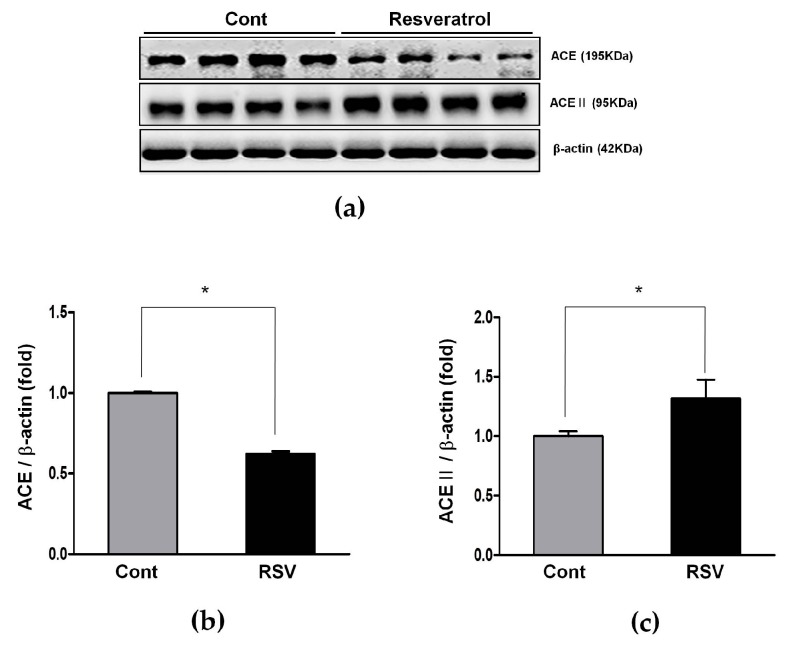
Effects of resveratrol on the angiotensin converting enzyme (ACE) and ACEII protein expressions. (**a**) Representative western blots of ACE and ACEII protein levels. (**b**) The protein levels of ACE were lower in the RSV group than in the control (Cont.) group. (**c**) The protein levels of ACEII were higher in the RSV group than in the control group. Quantitative analysis of the results is shown (* *p* < 0.05).

The expression of ACE, which converts Ang I to Ang II, was decreased significantly in the resveratrol-treated group, compared to the control group (control 1.00 ± 0.01 vs. resveratrol 0.62 ± 0.02-fold, Figure 4a,b).

**Figure 5:**
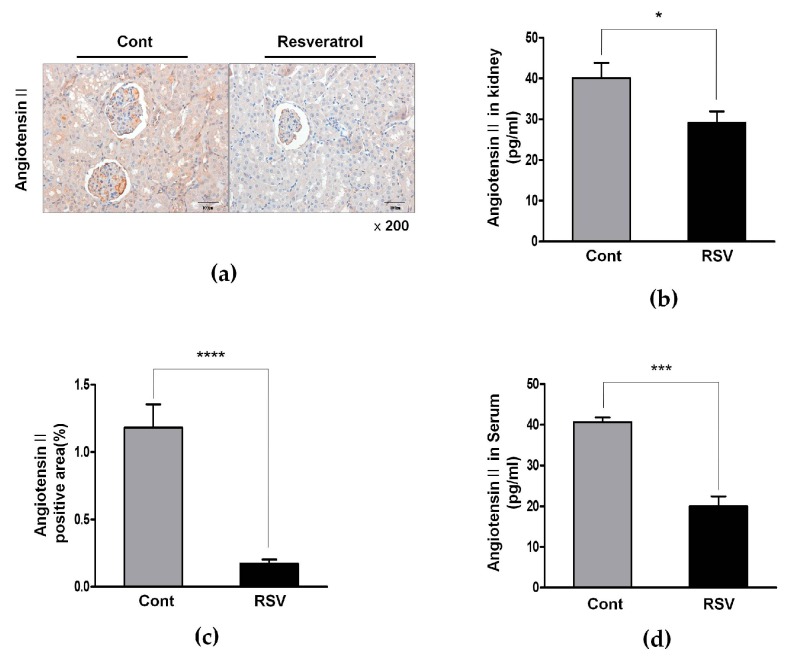
Effects of resveratrol on the Ang II. (**a**) Representative images of immunohistochemical staining with Ang II in aging kidney glomerulus (original magnification 200×). (**b**) The expression of Ang II in kidney was significantly decreased in the RSV group. (**c**) Ang II-positive area in kidney were observed to be significantly smaller in the RSV group; (**d**) The expression of Ang II in serum was also significantly decreased in the RSV group (* *p* < 0.05, *** *p* < 0.001, **** *p* < 0.0001).

Consequently, Ang II was also decreased in the resveratrol-treated group, compared with the control group; immunohistochemistry for Ang II showed decreased Ang II-positive areas (control 1.18 ± 0.49 vs. resveratrol 0.17 ± 0.08%, Figure 5a,c). enzyme immunoassay demonstrated significantly reduced renal Ang II levels (control 40.09 ± 3.76 vs. resveratrol 29.06 ± 2.85 pg/mL, Figure 5b) as well as decreased serum levels of Ang II (control 40.67 ± 1.16 vs. resveratrol 20.00 ± 2.43 pg/mL, Figure 5d).

**Figure 6:**
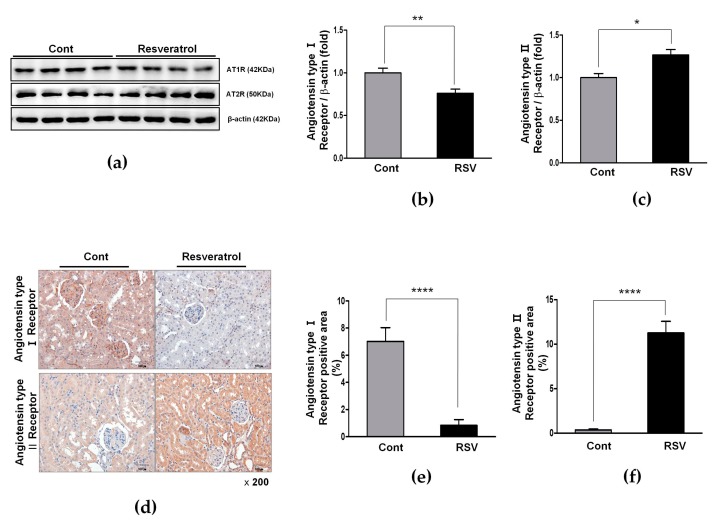
Effects of resveratrol on the AT1R and AT2R. (**a**) Representative western blots of AT1R and AT2R. (**b**) The expression of AT1R was significantly decreased in the RSV group. (**c**) The expression of AT2R was significantly increased in the RSV group. (**d**) Representative images of immunohistochemistry for AT1R and AT2R, in the aging kidney glomerulus (original magnification × 200). (**e**) The expression of AT1R-positive area in the kidney was markedly decreased in the RSV group. (**f**) The expression of AT2R-positive area in the kidney was markedly increased in the RSV group (* *p* < 0.05, ** *p* < 0.01, **** *p* < 0.0001).

Furthermore, the expression of AT1R was decreased significantly (control 1.00 ± 0.06 vs. resveratrol 0.76 ± 0.05-fold, Figure 6a,b), as well as AT1R-positive areas, by immunohistochemistry (control 7.02 ± 2.88 vs. resveratrol 0.84 ± 1.19%, Figure 6d,e).

### 3.4. Resveratrol Stimulates Angiotensin II Type 2 Receptors (AT2R) and Mas Receptor in Aging Mice

**Figure 7:**
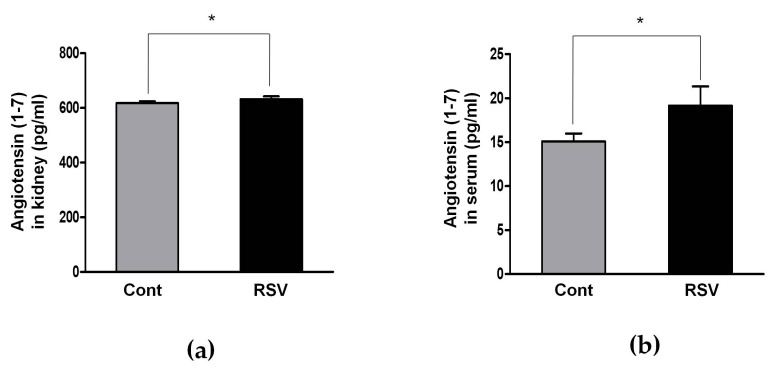
ELISA for serum and kidney levels of Ang 1-7. (**a**) Renal levels of Ang 1-7 significantly increased in the RSV group, compared to Cont. groups. (**b**) Serum levels of Ang 1-7 significantly increased in the RSV group, compared to Cont. groups. Quantitative analysis of the results is shown (* *p* < 0.05).

The RAS has another pathway comprised of ACE2, Ang 1-7, and MasRs that exerts a counter-regulatory function to Ang II activity. The expression of ACE2, which converts Ang II to Ang 1-7, was significantly increased in the resveratrol-treated group, compared to the control group (control 1.00 ± 0.04 vs. resveratrol 1.32 ± 0.16-fold, Figure 4a,c). Renal and serum levels of Ang 1-7 were analyzed by an enzyme immunoassay. Both the renal and serum levels of Ang 1-7 were significantly increased in the resveratrol-treated group, compared to the control group (control 618 ± 6 vs. resveratrol 632 ± 10 pg/mL, Figure 7a; and control 15.09 ± 0.91 vs. resveratrol 19.16 ± 2.18 pg/mL, Figure 7b; respectively).

**Figure 8:**
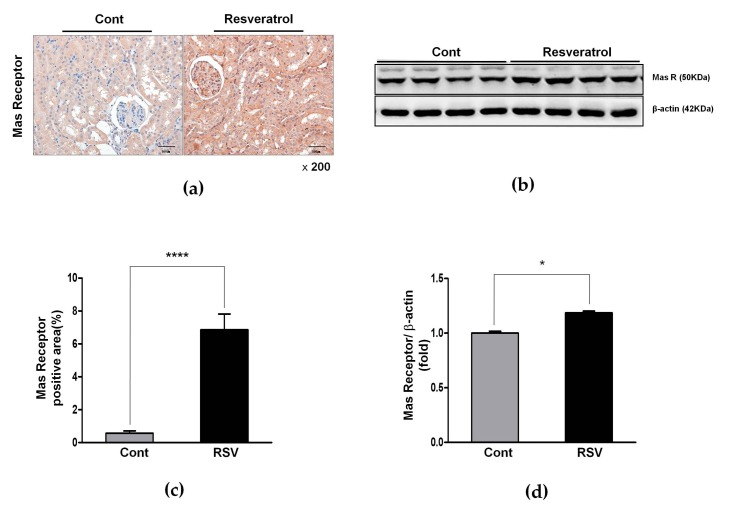
Effects of resveratrol on MasR. (**a**) Representative images of immunohistochemical staining of MasR in the aging kidney glomerulus (original magnification 200×). (**b**) Representative western blots of MasR. (**c**) MasR-positive area in the kidney was significantly increased in the RSV group. (**d**) The expression of MasR was significantly increased in the RSV group (* *p* < 0.05, **** *p* < 0.0001).

Accordingly, MasR, the effecter of Ang 1-7, was also significantly increased (control 1.00 ± 0.02 vs. resveratrol 1.19 ± 0.01-fold, Figure 8b,d).

The expression of AT2R, the negative regulator of AT1R, was increased significantly in the resveratrol-treated group, compared with the control group (control 1.00 ± 0.05 vs. resveratrol 1.27 ± 0.06-fold, Figure 6a,c). Immunohistochemistry for AT2R and MasR was performed and the results reinforced these findings. AT2R- and MasR-positive areas increased in the resveratrol-treated group (control 0.37 ± 0.27 vs. resveratrol 11.28 ± 1.28%, *p* < 0.001, Figure 6d,f; and control 0.57 ± 0.39 vs. resveratrol 6.86 ± 2.72%, *p* < 0.001, Figure 8a,c, respectively). These results showed that resveratrol inhibited the ACE/AT1R axis and that the ACEII/AT2R/MasR axis was activated.

### 3.5. Effects of the Resveratrol on the Oxidative Stress Marker

**Figure 9:**
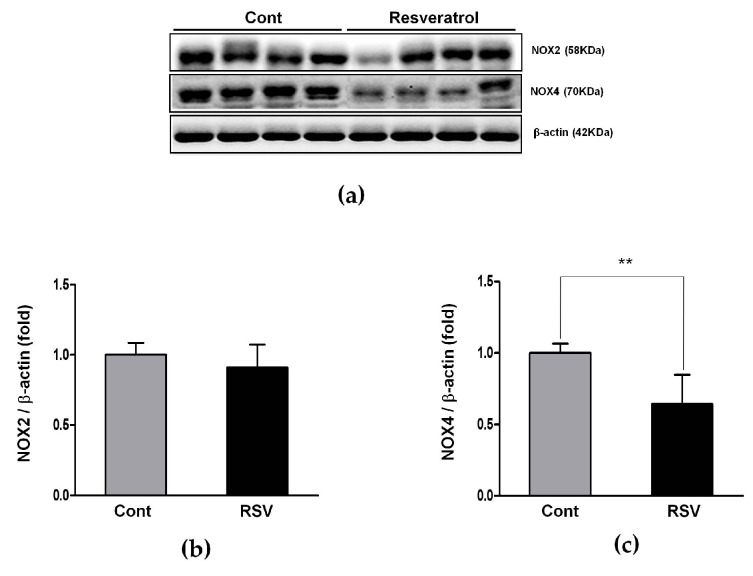
Effects of resveratrol on NOX2 and NOX4. (**a**) Representative western blots of NOX2 and NOX4 levels. (**b**) The expression of NOX2 showed a tendency of decrease in the RSV group, compared with the Cont. group, but it was not statistically significant. (**c**) The expression of Nox4 was significantly decreased in the RSV group. Quantitative analysis of the results is shown (** *p* < 0.01).

Previous reports show that oxidative stress and chronic exposure to ROS are key steps to age-related kidney changes [7,21,22]. Using western blot analyses, we examined the changes of the ROS generators NOX2 and NOX4. The expression of NOX2 tended to decrease without any statistical significance, while NOX4 was decreased significantly, in the resveratrol-treated group (control 1.00 ± 0.07 vs. resveratrol 0.64 ± 0.20-fold, Figure 9).

**Figure 10:**
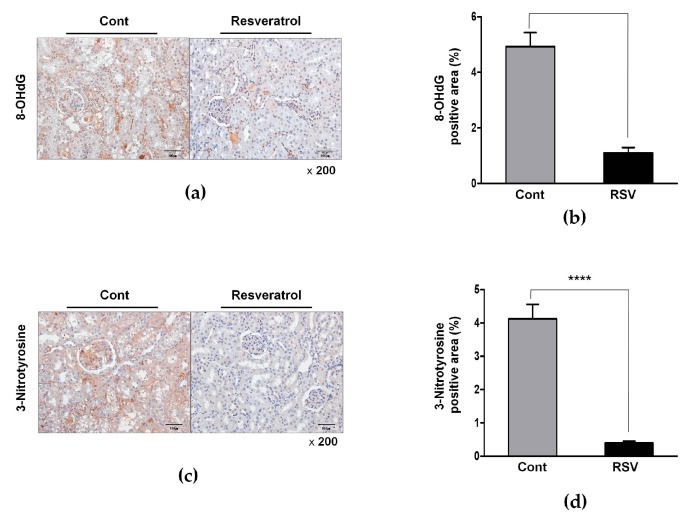
Effects of resveratrol on renal oxidative stress. (**a**) Representative images of immunohistochemistry for 8-OHdG in aging kidney glomerulus (original magnification 200×). (**b**) The positive area expression of 8-OHdG in the renal tissue was decreased in the RSV group, compared to that in the Cont. group. (**c**) Representative images of immunohistochemistry for 3-Nitrotyrosine in the aging kidney glomerulus (original magnification 200×). (**d**) The positive area expression of 3-Nitrotyrosine in the renal tissue was also decreased in the RSV group (**** *p* < 0.0001).

To evaluate changes in oxidative stress markers, immunohistochemical analyses of 8-OHdG and 3-nitrotyrosine were performed. The positive area of 8-OHdG (control 4.93 ± 1.41 vs. resveratrol 1.10 ± 0.55%, Figure 10a) and 3-nitrotyrosine (control 4.13 ± 1.22 vs. resveratrol 0.39 ± 0.17, Figure 10b) were significantly decreased in the resveratrol—treated group, compared with the control group. These findings indicated that resveratrol diminished the renal oxidative stress in the aging mice.

### 3.6. Effects of Resveratrol on the Antioxidant Enzyme

**Figure 11:**
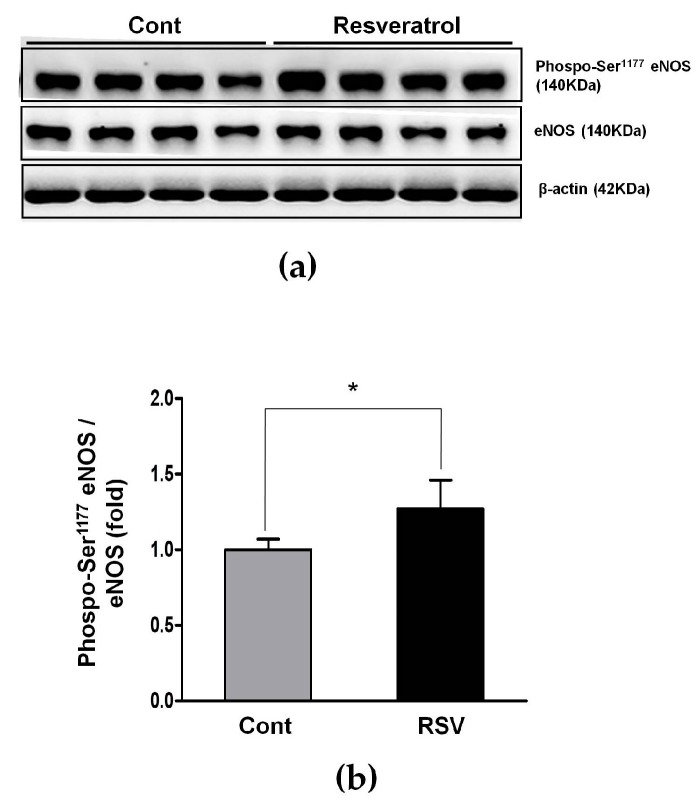
Effects of resveratrol on phospho-Ser1177eNOS/eNOS. (**a**) Representative western blots of phospho-Ser1177eNOS/eNOS levels. (**b**) The expression of phospho-Ser1177eNOS/eNOS was significantly increased in the RSV group. Quantitative analysis of the results is shown (* *p* < 0.05).

The influence of resveratrol on eNOS and the antioxidant enzymes SOD1 and SOD2 was examined by western blot analyses. The ratio of phospho-Ser1177 eNOS to the total eNOS was increased in the resveratrol-treated mice (control 1.00 ± 0.07 vs. resveratrol 1.27 ± 0.19-fold, Figure 11).

**Figure 12:**
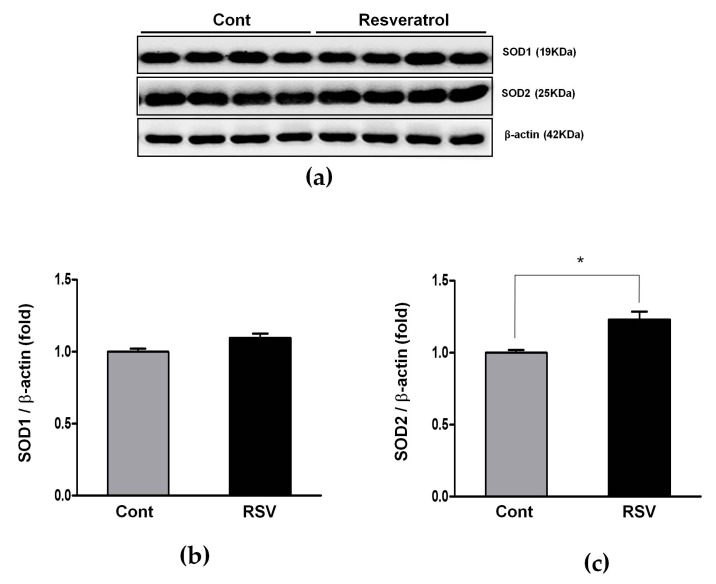
Effects of resveratrol on SOD1 and SOD2. (**a**) Representative western blots of SOD1 and SOD2 levels. (**b**) The expression of SOD1 showed a tendency of increase in the RSV group, compared with the Cont. group, but it was not statistically significant. (**c**) The expression of SOD2 was significantly increased in the RSV group. Quantitative analysis of the results is shown (* *p* < 0.05).

SOD2 protein levels were increased in the resveratrol-treated mice (control 1.00 ± 0.02 vs. resveratrol 1.23 ± 0.06-fold, Figure 12).

### 3.7. Anti-Inflammatory Effects of Resveratrol

**Figure 13:**
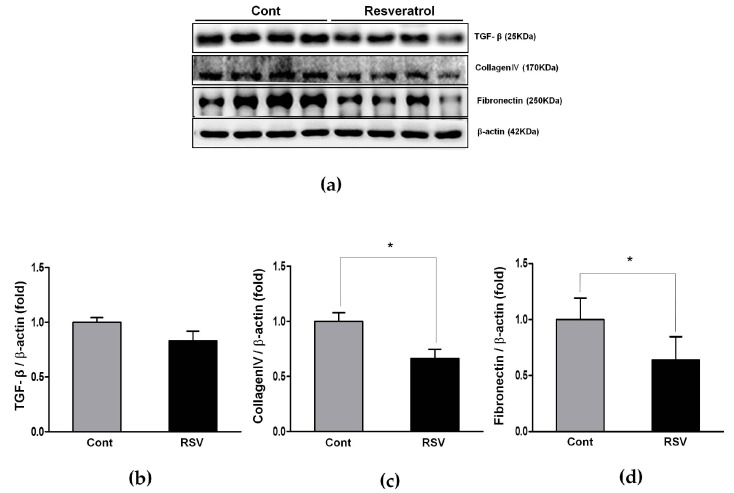
Effects of resveratrol on the TGF-β, collagen IV and fibronectin. (**a**) Representative western blots of TGF-β, collagen IV and fibronectin levels. (**b**) The expression of TGF-β showed a tendency of decrease in the RSV group, compared with the Cont. group, but it was not statistically significant. (**c**,**d**) The expression of collagen IV and fibronectin was significantly decreased in the RSV group. Quantitative analysis of the results is shown (* *p* < 0.05).

To evaluate the anti-inflammatory effects of resveratrol, we examined the expression of TGF-β, collagen IV, and fibronectin in kidneys, using a western blot analyses. TGF-β expression was decreased non-significantly in the resveratrol-treated group. The expression of collagen IV (control 1.00 ± 0.08 vs. resveratrol 0.66 ± 0.08-fold) and fibronectin (control 1.00 ± 0.19 vs. resveratrol 0.64 ± 0.21-fold) were decreased significantly by the resveratrol (Figure 13).

## 4. Discussion

In this study, we investigated the beneficial effects of resveratrol on the kidneys of aging mice. A six-month treatment with resveratrol resulted in a reduced albuminuria, better renal function, and improved renal histological changes, including diminished tubulointerstitial fibrosis, glomerulosclerosis, and inflammatory cell infiltration. Positive changes of the RAS were also observed in the resveratrol-treated aging mice. Specifically, the expression of ACE, Ang II, and AT1R was suppressed, while the expression of ACE2, Ang 1-7, AT2R, and particularly, MAS was stimulated. Furthermore, the expression of oxidative stress markers (NOX4, 8-OhdG, 3-nitrotyrosine) and inflammation markers (collagen IV, fibronectin) was improved in the resveratrol-treated mice. The novel finding of the present study is that resveratrol not only inhibits ACE, Ang II, and AT1R, the well-known classic axis, but also stimulates the ACE2/Ang 1-7/MasR axis in the aging kidneys. The altered expression of RAS components was confirmed by the western blot, as well as immunohistochemical assays.

Aging is a complex, multifactorial process, characterized by gradual deterioration of function and progressive structural changes [6,7]. Various possible mechanisms for aging and consequent anti-aging therapies have been studied including the free radical theory, immunological theory and mitochondrial theory, but the exact mechanism is still obscure [2,23,24,25]. Among potential mechanisms of aging, changes in the RAS, especially activation of the Ang II axis and inhibition of the MasR axis, are particularly important [26,27]. Previous reports have shown that chronic RAS activation promotes end-organ damage associated with aging [9,28] and the RAS blockade protects against renal aging caused by increasing the tissue and mitochondrial oxidative stress [29,30].

Recently, multiple lines of evidence suggested an association between an increased ACE2 signaling and improvements in the aging-related tissue injury. First, activation of ACE2 improved metabolic profiles during aging [31]. Second, endothelial dysfunction with aging was augmented in the ACE2-deficient mice [32]. Third, administration of an Ang 1-7 analogue was associated with improved aging-related neuroinflammation [33].

Numerous studies have reported that aging induces changes in the RAS [26,27]. For example, the baseline plasma renin level decreases and plasma renin activity is reduced with aging, which is associated with a reduction in both renin synthesis and release in the juxtaglomerular apparatuses [34,35]. This suppression of the systemic RAS leads to an impaired response to RAS stimuli, as well as to RAS inhibition [36]. Despite the decline in RAS activity, local secretion of Ang II, in the aging kidney, is markedly increased [37] and responsiveness to Ang II is enhanced [38]. In contrast, the ACE2/Ang 1-7/MasR axis is reduced with aging. Decreased expression of ACE2 and MasR is observed in the aortas of aging mice [39] and lower Ang 1-7 levels, in the mouse brains, are noted during aging [31]. Together, these events lead to age-associated functional and structural changes in the kidney. Many studies searching for anti-senescence antioxidants have been conducted. Resveratrol, a polyphenolic compound that occurs in many plants and plant products with potent antioxidant and anti-inflammatory activities, is among the numerous agents tested [13,40,41]. Resveratrol is a known activator of the AMP-activated protein kinase and SIRT1, which downregulate the expression of AT1R [42,43,44,45]. Previously, Miyasaki et al. reported that, in rat vascular smooth muscle cells, resveratrol downregulated the expression of AT1R via the activation of SIRT1 [46]. While Kim et al. revealed that inflammation, fibrosis, and oxidative stress in the aging aorta were attenuated by a resveratrol treatment, through regulation of the systemic and tissue-specific RAS [47]. Similarly, the results of the present study showed that resveratrol treatment attenuated oxidative stress, inflammation and fibrosis in the aging kidney, and modulated age-associated changes in the RAS components, by suppressing the PRR/ACE/Ang II axis and activating the ACE2/Ang 1–7/AT2R/MasR axis, in the mouse kidney.

Many of the molecular and cellular effects of Ang II are mediated by stimulating the production of ROS. Of the many types of ROS generated, NOX is considered as the prime producer [48,49]. To date, seven isoforms of NOX have been identified (NOX1–5, dual oxidase 1 and 2). NOX1, NOX2, and NOX4 are found in the kidney. NOX4 is the predominant renal form, whereas NOX1 and NOX2 have less functional importance [50,51]. Ang II is a potent stimulator of NOX. It activates the enzyme, increases expression of NOX subunits, and stimulates superoxide production [52,53,54]. Increased NOX activity and overproduction of mitochondrial ROS underlie the oxidative stress associated with aging and promote inflammation and tissue damage [55,56,57]. To date, numerous studies have been conducted about the organ-protective effect of the ACE2/Ang 1-7/MasR axis, through NOX suppression. Lo et al. reported that an infusion of the recombinant ACE2, lowered plasma Ang II, and increased the plasma Ang 1-7 levels, resulting in a significantly reduced NOX4, along with a lowered blood pressure [58]. In addition, Tanno et al. found that AT1R blockade enhanced the ACE2/Ang 1-7/MasR axis and suppressed the NOX4 expression, resulting in improved cardiac hypertrophy [59]. Our results congruently noted that a suppressed NOX4 expression, after the resveratrol treatment, was accompanied by an activation of the ACE2/Ang 1-7/MasR axis.

## 5. Conclusions

Our results demonstrated that the resveratrol treatment improves kidney function, albuminuria, glomerulosclerosis, tubular interstitial fibrosis, inflammation, and oxidative stress of age-related renal injury. These changes occur through decreases in the PRR/ACE/Ang II/AT1R axis and increases in the ACE2/Ang 1-7/MasR axis. A therapeutic strategy targeting the ACE2/Ang 1-7/MasR axis with resveratrol may postpone age-related renal structural and functional deterioration, through its antioxidant and antifibrotic effects. However, additional clinical investigations of the safety and anti-aging efficacy of resveratrol, are necessary.
